# Facile Synthesis of Low-Dimensional and Mild-Alkaline Magnesium Carbonate Hydrate for Safe Multiple Protection of Paper Relics

**DOI:** 10.3390/molecules29204921

**Published:** 2024-10-17

**Authors:** Yi Wang, Zirui Zhu, Jinhua Wang, Peng Liu, Xingxiang Ji, Hongbin Zhang, Yi Tang

**Affiliations:** 1 State Key Laboratory of Biobased Material and Green Papermaking, Qilu University of Technology, Shandong Academy of Sciences, Jinan 250353, China; ebanwang@163.com (Y.W.); 22110820002@m.fudan.edu.cn (Z.Z.); liupengfdu@fudan.edu.cn (P.L.); xxjt78@163.com (X.J.); 2 Department of Cultural Relics and Museology, Fudan University, Shanghai 200433, China; jinhuawang@fudan.edu.cn; 3 Institute for Preservation and Conservation of Chinese Ancient Books, Fudan University Library, Fudan University, Shanghai 200433, China; 4 Department of Chemistry, Laboratory of Advanced Materials, Collaborative Innovation Center of Chemistry for Energy Materials and Shanghai Key Laboratory of Molecular Catalysis and Innovative Materials, Fudan University, Shanghai 200433, China

**Keywords:** low-dimensional materials, mild alkalinity, protection of paper relics, safe deacidification, paper enhancement

## Abstract

Paper-based cultural relics inevitably face a variety of diseases such as acidification, yellowing, and strength loss during long-term preservation, where weakly alkaline inorganic materials play an important role in their deacidification treatments. In this work, by simply adjusting the supersaturation of crystal growing solution without the use of any organic additives, one-dimensional (1D) and two-dimensional (2D) weakly alkaline materials—magnesium carbonate hydrates (MCHs)—were controllably synthesized. It is worth noting that the coatings of 1D/2D MCHs not only cause little change in chromatic aberration and water wettability, but also ensure their safety for alkali-sensitive pigments. Meanwhile, the deacidification, anti-aging, strength-enhancing, and flame-retardant effects of these materials have been tested on ancient book papers, all of which achieved good protective effects. In contrast, 1D MCH materials brought about significant enhancement in both mechanical strengths and flame-retardant effects, and the related effects were investigated. Based on this facile micromorphology control strategy, more low-dimensional nanomaterials are expected to be synthesized by design for the protection of paper-based relics, which will expand our knowledge on functional deacidification and protection mechanisms.

## 1. Introduction

As one of the most important carriers of cultural information, paper-based relics play a vital role in the history of mankind. There exists inestimable historical, artistic, and scientific value in paintings, calligraphy works, archives, books, and other kinds of paper-based cultural relics. Although much importance has been attached to these precious tangible cultural heritages, deterioration unavoidably occurs during their storage. Naturally, as the main component of paper, cellulose undergoes a chain degradation reaction inevitably, which leads to the acidification of paper, causing yellowing, discoloration, strength decline, and other problems that threaten the longevity of paper relics [1,2]. To avoid the adverse consequences of accumulating acidity, deacidification has been considered the most crucial step in conservation of paper relics. Studies show that hydroxides or oxides are mainly employed as deacidification agents (such as calcium hydroxide [3,4,5] and magnesium oxide [6,7,8]). However, the excessive alkalinity of deacidification materials and the iterative treatment process may bring some side effects, causing damage to the fibers and thus reducing the mechanical properties of papers, which obviously go against the safety requirements for fragile paper relic protection [9,10]. Therefore, the mild alkaline, efficient, and multifunctional protective material is still desirable.

In recent decades, low-dimensional materials and their composites have received great research attention in terms of cellulose-based material enhancement and multi-functionalization [11,12,13] due to their diverse properties, and are expected to play a special role in paper relic protection. One-dimensional (denoted as 1D) materials usually exhibit typical cross-sectional dimensions in the nanoscale and lengths spanning from hundreds of nanometers to millimeters. Among them, whiskers are a special class of one-dimensional materials, which are short fibers grown from single crystals with a highly ordered atomic arrangement and fine diameters, and whose strength is close to the theoretical value of a complete crystal [14]. As in the paper industry, magnesium salt whiskers have been used as a filler to enhance the flame-retardant performance of paper, and calcium carbonate whiskers were proved to be effective in improving the physical properties of paper [15,16]. Two-dimensional (denoted as 2D) nanomaterials are layered compounds with a nanometer-scale thickness, large specific surface area, and a high density of surface-active sites capable of superior flexibility, transparency, and breathability. Existing research suggests that two-dimensional zeolite materials can not only provide sufficient, mild alkaline sites, but also improve the mechanical strength of the paper through film formation [17]. Due to the superior specific surface area and tunable surface chemistry, 2D materials can provide continuous protection as a good carrier, which helps to enhance the aging resistance of paper-based artifacts [18,19,20]. Nevertheless, although these low-dimensional synthetic materials exhibit excellent properties in paper-based materials, their synthesis process is complex and time-consuming, and organic templates and capping agents are usually used, which may limit their widespread use in the protection of paper relics.

Perhaps the simplest and most cost-effective manner to prepare low-dimensional inorganic materials is through precipitation from a solution. This process involves a nucleation and a growth stage. As the nuclei formed approach a critical concentration, additional reactants can either attach to the existing nuclei, leading to particle growth, or form new nuclei [21,22]. Naturally, the morphology of the precipitates produced depends on which of these two competitive steps prevails, although they all proceed quickly, the growth of particles and materials with the desired morphology can be facilitated by finding reasonable control conditions. Such synthetic methods to control the product morphology by adjusting the degree of ionization and supersaturation have also been reported in the literature in recent years [23]. Thus, based on this type of method, we expect to see more development of weakly alkaline materials for the protection of paper-based artifacts.

**Scheme 1:**
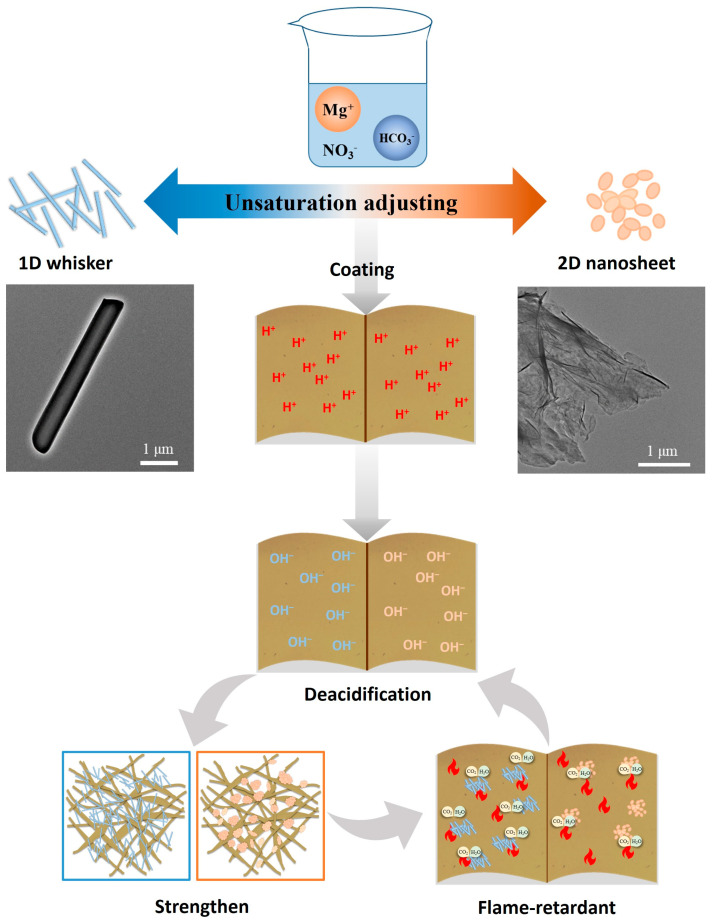
Illustration of micromorphology modulation of materials and their protection effect on paper relics.

Among the weakly basic inorganic compounds with tunable micromorphology, magnesium carbonate hydrates are a class of important materials, and the general formula can be expressed as xMgCO_3_·yMg(OH)_2_·zH_2_O (x = 1–5, y = 0–1, z = 1–8). To date, a number of synthesis methods have been developed to generate diverse morphologies [24,25,26,27]. However, the simple and low-cost controllable synthesis of low-dimensional magnesium carbonate hydrate materials still needs to be developed, and its new applications such as paper relic protection are not yet covered. In this paper, low-dimensional weakly alkaline magnesium carbonate hydrates with different morphologies (1D whisker and 2D nanosheet) were prepared by adopting a synthetic strategy of controlling crystal growth through solution supersaturation without using any organic additives, and the materials were tested for the coating effect and safety, as well as for their protective effects on historical wood-pulp papers (denoted as HMP). Both protective materials showed good deacidification and anti-aging properties, and their different structure and composition enabled them to play differentiated roles in the mechanical strength enhancement and flame-retardant performance improvement of paper. As shown in Scheme 1, this work provides a brand new perspective on the study of paper conservation materials and shows bright prospects in the protection of diverse cultural relics.

## 2. Results and Discussion

### 2.1. Structure and Properties of 1D and 2D Products

**Figure 1:**
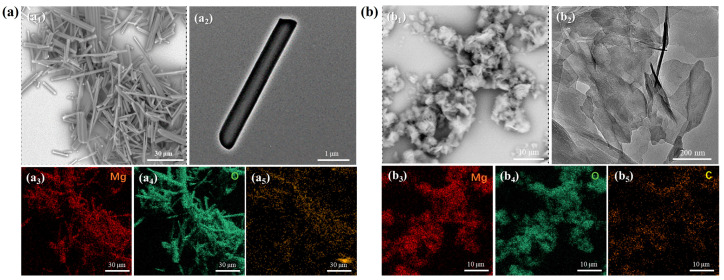
(**a_1_**) SEM image, (**a_2_**) TEM image, and (**a_3_**–**a_5_**) related element analysis of 1D product; (**b_1_**) SEM image, (**b_2_**) TEM image, and (**b_3_**–**b_5_**) related element analysis of 2D product.

The SEM and TEM results (Figure 1) clearly demonstrate that the synthesis method employed in this work easily yields good 1D and 2D microstructure. The diameter of 1D whisker is mainly between 0.5 and 1 μm, and the length can be larger than 30 μm. It can be seen from Figure 1(b_2_) that the thickness of 2D nanosheets is very thin, about 5 nm. The related element mapping data preliminarily indicate that the elemental composition of the two products is characteristic of the magnesium carbonate hydrate compounds. This result accords with the expectation of supersaturation adjustment design.

**Figure 2:**
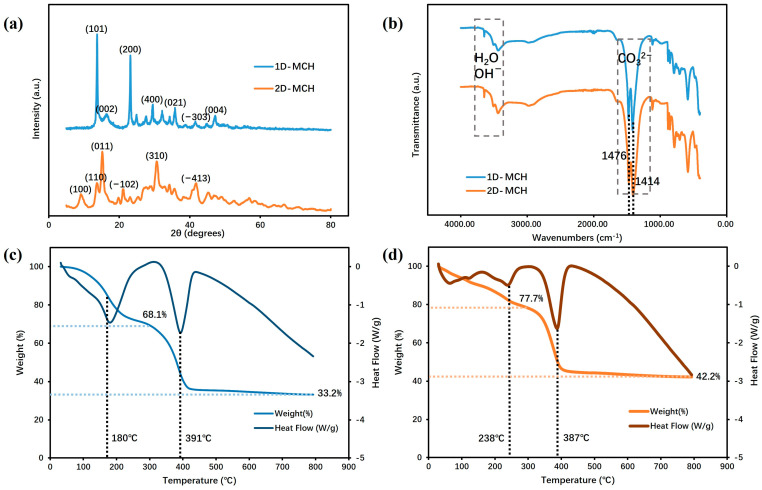
(**a**) XRD pattern and (**b**) FT-IR spectra of 1D and 2D products; (**c**) DSC-TGA curve of 1D product and (**d**) DSC-TGA curve of 2D product.

The XRD pattern (Figure 2a) reveals that both products with different morphologies are typically magnesium carbonate hydrates, and the phases of 1D and 2D products are MgCO_3_·nH_2_O and 4MgCO_3_·Mg(OH)_2_·nH_2_O, respectively [28,29], denoted as 1D-MCH and 2D-MCH. The peak style of the (101), (200), (400), (021), and (004) characteristic crystal faces of 1D-MCH are sharp, indicating the good crystallinity of the sample. However, the diffraction peaks corresponding to the characteristic crystal plane (100), (011), (−102), (310), and (−413) of 2D-MCH are relatively wide, influenced by the small size of the sample layer. Further, the FT-IR data are tested to study the state of characteristic groups inside these samples. The absorption peaks at 3200–3500 cm^−1^ in the IR spectrum (Figure 2b) are characteristic of water and -OH in the sample, while the stronger double peaks at 1476 and 1414 cm^−1^ are asymmetrical contraction vibrations of the carbonate, and the weaker absorption at 1117 cm^−1^ is the symmetrical stretching vibration of the carbonate. The similar characteristic regions in the IR spectra of the two samples indicate that they have a close composition.

In addition, a thermogravimetric analysis (TGA) is adopted to explore the thermal decomposition and compositions of these samples, as shown in Figure 2c,d. 1D-MCH is essentially stable in a mass up to 100 °C, with its first peak in the DSC curves at 180 °C due to the loss of crystal water, and the mass loss in this stage is 32.1%. In contrast, 2D-MCH is less thermally stable at low temperatures (<100 °C), with weight loss and endothermic in the early stage of heating, with 22.3% of mass loss in the first stage. The highest peak of DSC curves of 1D-MCH and 2D-MCH was observed at 391 and 387 °C, respectively, indicating the release of CO_2_. Thus, in the second stage, the mass loss for 1D-MCH and 2D-MCH is 34.7% and 35.5%, respectively, which is jointly composed of contributions from the release of carbon dioxide and water. At about 400 °C, the mass percentage of the two samples gradually tends to be stable, and finally, they will completely decompose into MgO. Based on the final mass percentage, it can be calculated that the average number of water molecules bound in 1D-MCH and 2D-MCH crystals is 2.1 and 4.6, respectively.

### 2.2. Coating Property and Safety Test

**Figure 3:**
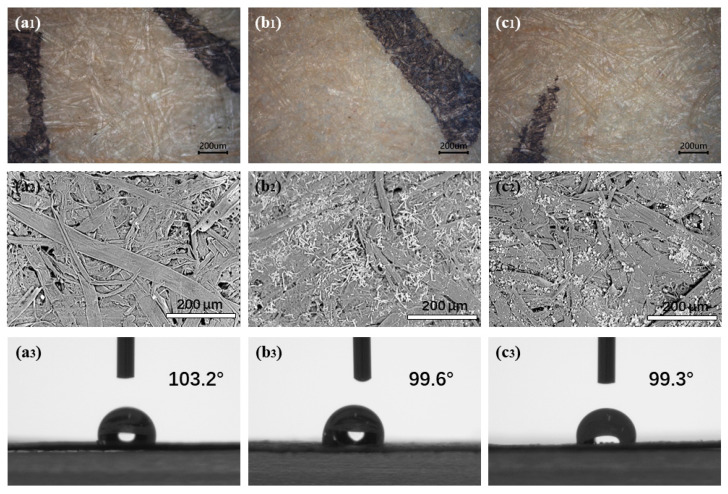
(**a_1_**) Optical microscopy image, (**a_2_**) SEM image and (**a_3_**) contact angle of water droplets of uncoated HMP; (**b_1_**) Optical microscopy image, (**b_2_**) SEM image and (**b_3_**) contact angle of water droplets of 1D-MCH-coated HMP; (**c_1_**) Optical microscopy image, (**c_2_**) SEM image and (**c_3_**) contact angle of water droplets of 2D-MCH-coated HMP.

Before the application experiments, several tests were performed to evaluate the coating property and safety of protective materials, including the contact angle tests of the paper samples before and after coating and chroma changes during dry-heat aging. It can be seen from Figure 3 that the deposition of material formed a good microscopic filling between the paper fibers. Especially in the 1D-MCH-treated sample, the filling compactness between fibers was significantly improved. At the same time, the contact angle of paper samples had no significant change, which indicates that the treated paper still maintains a certain hydrophobicity. Furthermore, the results of the chromaticity change show that the effect of protection treatment on paper chromaticity is in an acceptable range (Table 1).

Among the various international conventions on the protection of cultural heritage, integrity and authenticity and are two main principles to be followed in the protection of tangible cultural heritage. When it comes to paper relics like paintings and calligraphy relics, one of the most important original states is the color of the pigments used. Therefore, it is an important indicator to evaluate the safety of alkaline deacidifiers used in paper deacidification protection that whether they will adversely affect the color of alkali-sensitive pigments. In this work, two pigments sensitive to alkali, lead chrome yellow (PbCrO_4_) and Prussian blue (Fe_4_[Fe(CN)_6_]_3_), were selected to test the influence of alkaline protection materials on their color.

**Figure 4:**
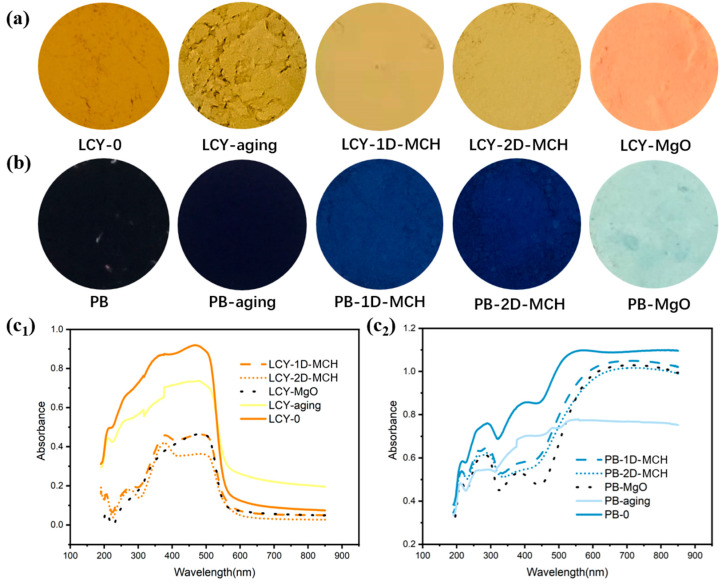
Photographs (**a**,**b**) and UV–Vis spectra of lead chrome yellow(**c_1_**) and Prussian blue (**c_2_**), mixed with 1D-MCH, 2D-MCH, and MgO.

As shown in Figure 4a, the color state of lead chrome yellow (LCY) changed slightly after accelerated aging. The study by Costana et al. proved that the reduction in Cr(VI) to Cr(III) caused by light should be the main reason for the aging and fading of lead chrome yellow in oil painting, which is also related to the purity of lead chromate in pigment [30]. However, as an important mineral pigment widely used in the Chinese art of painting during the Qing Dynasty (A.D. 1636–1921), Prussian blue (PB) showed no obvious color changes after aging (see Figure 4b). However, it can be seen in Figure 4(c_2_) that there is a significant intensity change in the UV absorption band. Therefore, the visual color maintenance may be attributed to the strong tinting ability and high saturation of the pigment.

After being mixed with the protective materials, the intensity of UV absorption band for each sample decreased because of the diluting effect of material powders (Figure 4(c_1_,c_2_)), which can be avoided to the greatest extent by adjusting the dosage and action mode of protection materials. For 1D-MCH- and 2D-MCH-treated samples, the type and position of the peaks in UV absorption bands remain unchanged, indicating that the protective material did not damage the color mechanism of the pigments. In contrast, commercial MgO had a serious effect on the color of LCY and PB. The UV curve changed obviously, and the color of LCY turned red, while PB almost completely faded. This kind of undesirable consequence may be due to the formation of more alkaline Mg(OH)_2_ in the MgO dispersion, while both LCY and PB are very unstable under alkaline conditions and present different colors with different oxidation states. Therefore, for paper relics using alkali-sensitive pigments, it is safer to use weakly alkaline 1D-MCH and 2D-MCH than commercial MgO as deacidification agents.

### 2.3. Deacidification and Anti-Aging Properties

Acidification is one of the main diseases threatening the life of paper-based cultural relics, and thus, deacidification is crucial in extending the life span of paper relics. It should be noted that the goal of deacidification is not only to neutralize the acid already produced in the paper but also to deposit an alkaline substance to neutralize the acid that may be produced in the future, that is, to form a long-term alkaline reserve in cultural relics. Therefore, in addition to comparing the pH value before and after deacidification, the pH change in different treated paper samples during dry-heat aging was also tracked.

Before coating, the surface pH value of HMP was 4.07, reflecting that the paper was at a serious acidification level. After coating with three materials, respectively, the pH of the paper samples increased significantly. *The technical specifications and quality requirements for the restoration of ancient books* formulated by the National Museum of China stipulate that the paper after deacidification must be in the neutral or weakly alkaline region, which means the pH should between 7.5 and 10. Among the deacidification materials and the control groups used in this work, all three could keep the alkalinity of the paper samples within the desired range.

**Figure 5:**
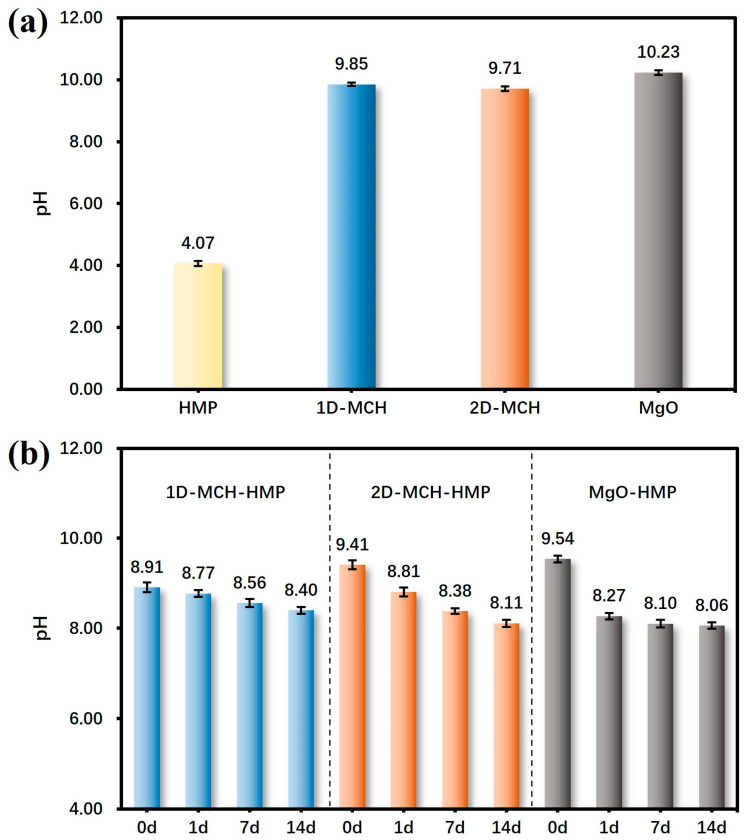
(**a**) The pH of HMP paper samples before coating and the dispersion of various materials (0.100 g material in 9.900 g 75 % ethanol); (**b**) the pH of coated samples undergoing dry-heat aging.

After 14 days of dry-heat aging, the pH value of each sample decreased to a certain extent, as Figure 5b displays. Although the results of the tests immediately after coating showed that the alkalinity of the 1D-MCH-coated samples were not highly alkaline, with a pH below 9, after 14 days of dry-heat aging, the pH of this group of specimens remained at a high level, which indicates that the material has a good durability in terms of deacidification. In comparison, the 2D-MCH-treated samples showed a mediocre alkaline persistence, which may be related to the stability of the alkali carbonate. However, the pH of the commercial MgO-treated samples showed a large change from the first day of accelerated aging, decreasing from 9.54 to 8.27. After 14 days, the pH of this group of specimens was the lowest, indicating that it has a quick- rather than slow-release action.

### 2.4. Strengthening Performance, Flame-Retardant Property, and Mechanism

Combining the results of safety test and deacidification experiments, it can be seen that although the alkalinity of 1D-MCH and 2D-MCH is lower than MgO, their long-term deacidification effect is better than that of MgO, and both of them are safe choices. The performance of the material’s deacidification ability should be mainly attributed to its chemical composition, while the different structures of the two materials are expected to play a more differentiated role in the physical properties. Therefore, after the deacidification and anti-aging experiments, the different structural properties of 1D-MCH and 2D-MCH were further explored in the enhancement of paper mechanical properties.

**Figure 6:**
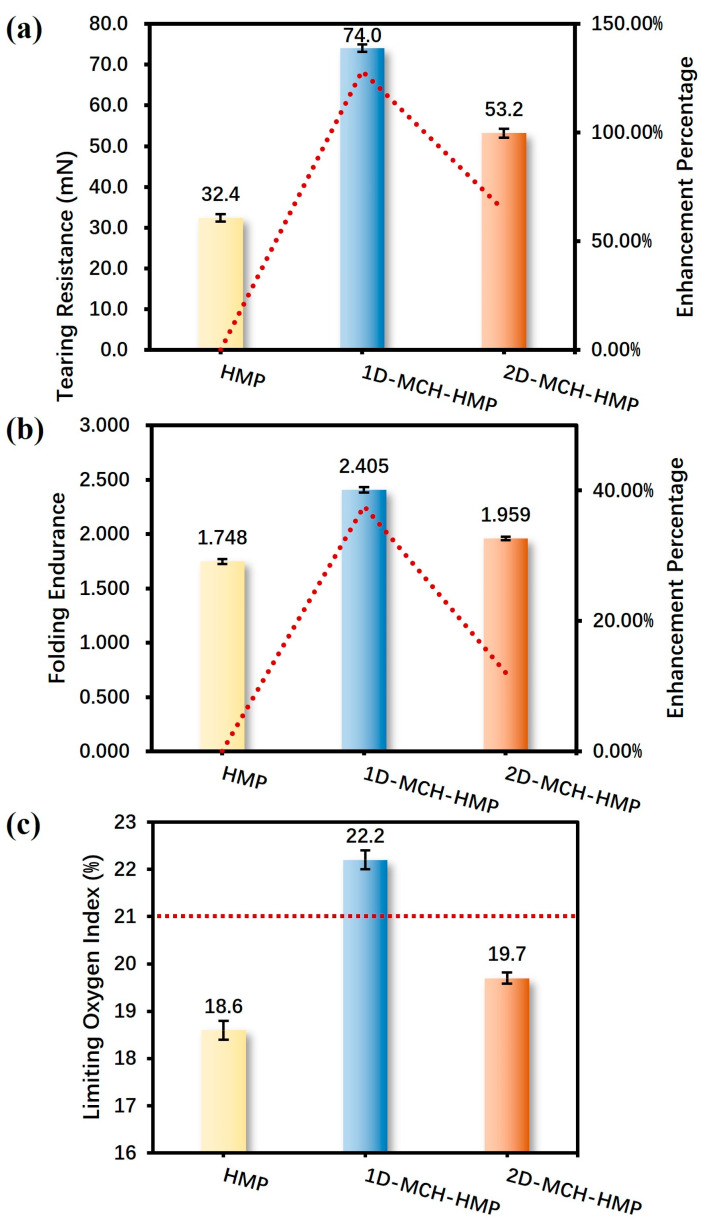
(**a**) Tearing resistance and enhancement percentage (red line) of different samples; (**b**) folding endurance and enhancement percentage (red line) of different samples; (**c**) limiting oxygen index of different samples.

The results of tearing resistance and folding endurance tests of the paper samples before and after coating show that both protective materials have an excellent strengthening effect on paper, especially in the enhancement of tearing resistance (Figure 6), which increased by ca. 128.4% (1D-MCH) and 64.2% (2D-MCH), respectively, compared with uncoated paper. It is obvious that 1D-MCH is better than 2D-MCH in improving the mechanical properties of paper relics, although the latter one can already achieve good results. Further, the test results of the ultimate oxygen index show that both types of low-dimensional materials with different morphologies can have a certain flame retardant effect on HMP (Figure 6c). The limit oxygen index of 1D-MCH is higher than that of 2D-MCH, reaching 22.2%, which is higher than the oxygen content in the air (about 21%, marked with the red dashed line in Figure 6c). This indicates that HMP loaded with 1D-MCH is difficult to burn stably in air, and the material can achieve good flame retardant protection effect.

**Figure 7:**
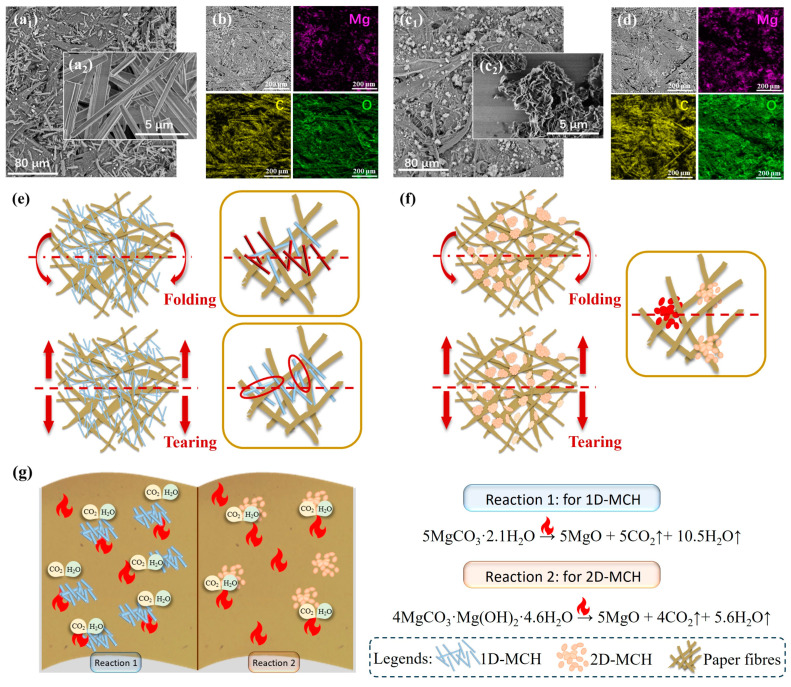
(**a_1_**,**c_1_**) SEM images of paper samples coated by 1D-MCH and 2D-MCH; (**a_2_**,**c_2_**) FESEM images of 1D-MCH and 2D-MCH; (**b**,**d**) SEM images and related Mg, C, and O mapping images of paper samples coated by 1D-MCH and 2D-MCH; (**e**–**g**) schematic diagrams of 1D-MCH and 2D-MCH deposition in paper fibers and their reinforcement and flame retardant mechanisms.

The SEM images and related element analysis results confirm that through the coating procedure, the protective materials can deposit uniformly in the paper fibers and could serve to fill the voids between fibers (Figure 7a–d), even when the fibers of HMP were flattened during its production process. The good coating condition provides the possibility for low-dimensional materials to play the role of strengthening and flame retardancy.

Further analysis of the protective mechanisms of two different low-dimensional materials in HMP reveals that the reinforcement effect of materials is not only related to the filling density but also depends on the mechanical strength of the material itself and the mode of action between protection materials and paper fibers.

As a kind of single crystal fiber, one-dimensional whisker material is very small in diameter, so it is difficult to accommodate internal defects in large crystals, and thus, its strength is close to the theoretical value of complete crystals. As illustrated in Figure 7e, for 1D-coated samples, the deposition of 1D-MCH not only fills the fiber gaps and increases their density, but also provides some support when the paper is bent. Thanks to the high strength of the whiskers, this one-dimensional deposit can counteract part of the impact of external forces to protect the paper fibers, thus significantly improving their folding resistance. In addition, when the paper is subjected to tearing tension, the one-dimensional material can act as a bridge between the paper fibers, enhancing its resistance to tearing.

In contrast, the filling of two-dimensional materials, while also strengthening the fragile paper, is not as effective as one-dimensional materials. Although theoretically, the flexibility brought by the nanosheets’ extremely thin size can help the material to fill in the gaps between the paper fibers, the SEM image and related element analysis results revealed that 2D-MCH failed to be distributed as layers in the paper. Instead, the nanosheets formed flower-like aggregates and presented in fibers in a filling or adhesion way, see Figure 7c.

Meanwhile, the flame retardancy of the material is related to its composition and thermal stability. Both 1D-MCH and 2D-MCH belong to hydrated magnesium carbonate materials, which can decompose after being heated and release water and carbon dioxide to delay combustion, as shown in Figure 7g. It can be seen from the TGA results (Figure 2c,d) that the ratio of water and carbon dioxide released by 1D-MCH after complete decomposition is higher, and the loss of its bound water begins at 180 °C, while the decomposition of 2D-MCH begins below 100 °C. The relatively high thermal stability and high levels of water and carbon dioxide in the composition are both reasons why 1D-MCH is able to show better flame retardancy.

## 3. Experimental Section

### 3.1. Reagents and Materials

Magnesium nitrate hexahydrate (Mg(NO_3_)_2_·6H_2_O, analytical reagent (AR), Shanghai Chemical Co., Shanghai, China) and sodium bicarbonate (NaHCO_3_, AR, Sinopharm, Beijing, China) were used to synthesize products with different micro-morphologies. For protective tests, naturally aged handmade wood-pulp paper from a book published in 1949 (denoted as HMP) were used as paper samples. Lead chrome yellow (PbCrO_4_, AR, Shanghai Chemical Co., Shanghai, China, denoted as LYC) and Prussian blue (Fe_4_[Fe-(CN)_6_]_3_, AR, Shanghai Chemical Co., Shanghai, China, denoted as PB) were selected to test the safety of different protective materials. A commercial metallic oxide reagent, magnesium oxide (MgO, AR, Sinopharm, Beijing, China), was regarded as the reference sample for both deacidification and anti-discoloration tests. Deionized water and anhydrous ethanol were used throughout.

### 3.2. Methods

#### 3.2.1. Preparation of Low-Dimensional Materials

This paper adopts a convenient and energy-efficient supersaturation adjust method to obtain 1D and 2D materials. What Wei-Hong Lai and co-workers have previously done is to judiciously tune the reaction system by adjusting the electrolytic dissociation (α) of the precursors and the supersaturation (S) of the solution to favor the growth of specific morphologies, without the need for any additives [23]. Combining the methods reported in the literature, we used Mg(NO_3_)_2_ as the magnesium ion providing agent, and controlled the ionization degree of the reactants in the solution by the weak electrolyte NaHCO_3_, on the basis of which the degree of supersaturation of the solution was regulated in order to obtain the products with different microcosmography.

For the 1D product, 0.065 M Mg(NO_3_)_2_·6H_2_O was dissolved in 30 mL deionized water, heated to 80 °C, and stirred for 30 min at the speed of 300 rpm to obtain an even solution. Then, 0.198 M NaHCO_3_ was added into the solution as quickly as possible. The products of the reaction were collected by centrifugation after 5 min, washed with water three times and freeze-dried for three days. The supersaturation of the system was adjusted to obtain the 2D product, in which 0.130 M Mg(NO_3_)_2_·6H_2_O and 0.600 M NaHCO_3_ were used, while the other steps remained unchanged.

#### 3.2.2. Coating Procedure

Protective materials were deposited on paper samples through a dip-coating method. The paper samples were cut into several sizes of specimens (2 × 2 cm^2^ for accelerated-aging, 5.0 × 6.5 cm^2^ for tearing test, 1.5 × 10 cm^2^ for folding endurance test, and 14 × 5.2 cm^2^ for flame retardancy test). Each paper sheet went through a round of 5 min immerse in 10 mL preconfigured protective suspension (0.100 g material in 9.900 g 75% ethanol) and 5 min naturally drying, performed 5 times in total. And then all the coated papers were dried under a vacuum condition at 25 °C for 24 h for subsequent testing.

#### 3.2.3. Dry-Heat Aging

The aging of paper samples was accelerated through an artificial dry-heat process by keeping them in a thermostatic chamber at 105 °C with air circulation (ISO 5630–1:1991) [31]. The paper samples were stabilized at 25 °C for at least 24 h before and after aging (ISO 187–1990) [32].

#### 3.2.4. Safety Test

To evaluate the safety of protection materials, the contact angle and chromaticity changes in the pattern were tested. Two alkali-sensitive pigments, PB and LCY, were selected for the safety test. In this experiment, 3 mL pigment dispersion (1 wt%) was mixed with 7 mL protective suspension. After being shaken for 5 min and heated in an oven at 80 °C for 24 h, the mixture was filtered and dried for the UV-Vis test.

#### 3.2.5. Protection Effect

The protection performance of materials was evaluated via deacidification and the enhancement of mechanical properties of samples after coating. The pH values of paper samples coated with different materials were compared to evaluate the deacidification effect of the materials. Then, on the 1st, 7th, and 14th days of accelerated aging, the paper samples were extracted for pH testing to judge the anti-aging properties of the protective agents. In terms of the enhancement effect of mechanical properties, the tearing resistance, folding endurance, and flame retardancy of the paper samples before and after coating were compared.

### 3.3. Characterization

Powder X-ray diffraction (XRD) was tested on a Bruker D2-Advanced diffractometer (Bruker Corporation, Billerica, MA, USA) from 5 to 80° with a scanning rate of 5° min^−1^. The related element analysis, morphologies, and structures were investigated by scanning electron microscopy (SEM, Phenom Prox, PhenomWorld, Eindhoven, The Netherlands), field emission scanning electron microscopy (FESEM, Nova NanoSEM 450, Thermo Fisher Scientific, Eugene, OR, USA), transmission electron microscopy (TEM, JEM 2010, JEOL Ltd., Tokyo, Japan), and field emission transmission electron microscopy (FETEM, Tecnai G2 F20 S-Twin, Thermo Fisher Scientific, Eugene, OR, USA). A thermogravimetric analysis (TGA) was performed on a SDT Q600 thermal analysis instrument (TA Instruments, Columbus, OH, USA) with a heating rate of 10 °C/min under air flow (100 mL/min). The UV-vis absorption spectra was obtained by a Lambda 650 spectrophotometer (PERKIN ELMER, Hopkinton, MA, USA). For the properties of the paper samples, the static contact angles were measured on an OCA15+ contact angle system (Dataphysics, Falkenhagen, Germany) at ambient temperature using a water drop volume of 5 μL, the pH values of paper samples were measured using a paper pH meter (HANNA HI 99171, HANNA Instruments, Sliema, Malta) with a planar electrode, and the chroma changes in the paper samples were analyzed using an NR10QC colorimeter (Konica Minolta, Tokyo, Japan). In terms of mechanical properties, the folding endurance was performed by referring to ISO 5626:1993 [33], and the tearing resistance was conducted according to ISO 1974:1990 [34] with a tension of 1.5 N. The flame retardancy of the sample was tested according to GB/T 2406.2 [35].

## 4. Conclusions

In this paper, we report the use of a facile synthesis strategy to obtain well-formed 1D and 2D MCHs, which were applied in experiments for the protection of paper-based cultural relics. The safety performance of the two materials was tested according to the special requirements of cultural relic protection, and the results showed that both materials did not lead to significant discoloration of paper, nor did they cause significant changes in the color of alkali-sensitive pigments. The results of the deacidification and aging experiments show that the deacidification effect of both materials can meet the requirements for the protection of paper cultural relics, and successfully form a long-term alkaline reserve in the paper samples, with a certain degree of anti-aging effect. In addition, thanks to its own structural properties, the 1D-MCH performs well in enhancing the mechanical strength of paper, and can bring about a flame-retardant effect, realizing the combination of safe deacidification protection and effective enhancement. In this study, the mild deacidification and anti-aging protection of paper cultural relics were realized from the synthetic modulation of material micromorphology, and the strength-enhancing effects of low-dimensional materials with different micromorphologies and their mechanisms were explored, which provided a new vision for further research on paper cultural relic protection materials.

## Figures and Tables

**Table 1:** CIE color coordinates of uncoated and coated HMP.

Sample (HMP)	L	a*	b*	ΔE*
uncoated	78.96	8.67	21.90	--
1D-MCH-coated	77.93	7.35	21.40	1.76
1D-MCH-coated, aged for 7 d	78.16	7.42	22.04	1.50
1D-MCH-coated, aged for 14 d	77.98	7.03	21.08	2.09
2D-MCH-coated	78.50	7.11	20.81	1.96
2D-MCH-coated, aged for 7 d	78.33	6.85	21.78	1.94
2D-MCH-coated, aged for 14 d	80.30	8.94	21.52	1.41

* These values are calculated based on the CIEDE2000 formulae.

## Data Availability

Data are contained within the article.
